# The Unfolded Protein Response in Amelogenesis and Enamel Pathologies

**DOI:** 10.3389/fphys.2017.00653

**Published:** 2017-09-08

**Authors:** Steven J. Brookes, Martin J. Barron, Michael J. Dixon, Jennifer Kirkham

**Affiliations:** ^1^Division of Oral Biology, School of Dentistry, University of Leeds, St James's University Hospital Leeds, United Kingdom; ^2^Faculty of Biology, Medicine and Health, Manchester Academic Health Sciences Centre, University of Manchester Manchester, United Kingdom

**Keywords:** ameloblast, ER stress, unfolded protein response, apoptosis, amelogenesis imperfecta, fluorosis

## Abstract

During the secretory phase of their life-cycle, ameloblasts are highly specialized secretory cells whose role is to elaborate an extracellular matrix that ultimately confers both form and function to dental enamel, the most highly mineralized of all mammalian tissues. In common with many other “professional” secretory cells, ameloblasts employ the unfolded protein response (UPR) to help them cope with the large secretory cargo of extracellular matrix proteins transiting their ER (endoplasmic reticulum)/Golgi complex and so minimize ER stress. However, the UPR is a double-edged sword, and, in cases where ER stress is severe and prolonged, the UPR switches from pro-survival to pro-apoptotic mode. The purpose of this review is to consider the role of the ameloblast UPR in the biology and pathology of amelogenesis; specifically in respect of amelogenesis imperfecta (AI) and fluorosis. Some forms of AI appear to correspond to classic proteopathies, where pathological intra-cellular accumulations of protein tip the UPR toward apoptosis. Fluorosis also involves the UPR and, while not of itself a classic proteopathic disease, shares some common elements through the involvement of the UPR. The possibility of therapeutic intervention by pharmacological modulation of the UPR in AI and fluorosis is also discussed.

## Introduction

Amelogenesis involves the incremental secretion of a self-assembling extracellular protein matrix (enamel matrix) on to the pre-existing dentine surface by columnar secretory ameloblasts. The enamel matrix is overwhelmingly (>90%) composed of proteins derived by extracellular proteolysis of alternatively spliced products of the amelogenin gene (*AMELX/Y* in humans; *Amelx* in rodents) (Brookes et al., 1995). Other, far less abundant, matrix proteins that are secreted during the secretory phase of amelogenesis include: enamelin (ENAM), ameloblastin (AMBN) and matrix metallopeptidase 20 (MMP20) (Moradian-Oldak, 2012; Bartlett, 2013). AMELX, ENAM, and AMBN are generally regarded as structural components of the enamel matrix whereas MMP20, present in catalytic amounts, is responsible for the proteolytic processing of AMELX, ENAM, and AMBN. Enamel is partially mineralized during the secretory phase. Extremely elongated crystallites of hydroxyapatite, originating at the enamel-dentine junction, grow in length (c-axis growth) surrounded by enamel matrix proteins that are newly secreted by the ameloblasts as they migrate away from the enamel dentine junction. Secretory stage ameloblasts have the typical characteristics of a specialized secretory cell, including numerous mitochondria and a well-developed endoplasmic reticulum (ER)/Golgi complex (Reith, 1961). These adaptations allow the ameloblasts to cope with their large secretory load as they incrementally secrete the enamel matrix.

Enamel crystallites are organized into bundles (the so-called enamel prisms or rods) which are interspersed with inter-prismatic enamel crystals that together delineate the enamel ultrastructure; a process directed by the ameloblasts' specialized Tomes' process from which the secretory cargo is elaborated (Smith, 1998). The function of the enamel matrix and its component parts is still not fully understood but the consensus view is that it is involved with the nucleation of the enamel crystallites, the control of their subsequent preferential c-axis growth, and their structural organization into prisms and inter-prismatic enamel. Once the ameloblasts have secreted the required thickness of enamel, matrix secretion ceases. The ameloblasts become shortened and less columnar and lose their Tomes' processes. This marks the end of the secretory phase and the beginning of the maturation phase (Smith, 1998) which is further characterized by ameloblasts up-regulating the expression and secretion of a number of maturation stage-specific proteins including kallikrein-related peptidase 4 (KLK4) (Bartlett, 2013), amelotin (AMTN) (Iwasaki et al., 2005), and odontogenic ameloblast-associated protein (ODAM) (Nishio et al., 2010). KLK4 is a serine protease that quickly degrades the spectrum of proteins comprising the secretory stage matrix, facilitating their ultimate removal from the tissue by ameloblast endocytosis (Lacruz et al., 2013). As the enamel matrix is removed, it is replaced by fluid into which the ameloblasts actively transport mineral ions which drive the growth of the enamel crystallites in width and thickness so that they eventually occlude most of the tissue volume.

The functional importance of the enamel matrix proteins in amelogenesis is evidenced by the effects of mutations in their respective genes on the enamel phenotype, which can result in amelogenesis imperfecta (AI), characterized by biomineralization defects of enamel (Smith et al., 2017). Several studies have examined the potential effects of such mutations on events occurring in the enamel extracellular matrix itself, including protein-protein interactions and enamel matrix self-assembly (Lakshminarayanan et al., 2010; Zhu et al., 2011) and also protein-mineral interactions (Zhu et al., 2011). Certainly, perturbation of these processes would be expected to give rise to enamel biomineralization defects and therefore AI, including, for example, a complete failure to produce enamel, the production of pathologically thin or under-mineralized enamel or enamel in which the ultrastructural arrangement of the crystallites is affected. However, recent data have suggested that intra-cellular events related to the so-called unfolded protein response (UPR) may also play an important role in enamel biology and pathology—including AI and fluorosis.

The UPR is a signaling pathway that has evolved to allow cells to manage their secretory load under normal physiological and pathological conditions to maintain proteostasis in the endoplasmic reticulum (ER) (Hetz et al., 2015). Failure to maintain proteostasis can lead to ER stress which is a factor in many diseases (Kopito and Ron, 2000; Ozcan and Tabas, 2012). Our aim is to review the literature detailing the way in which secretory ameloblasts cope with their large secretory burden by utilizing the UPR in a similar way to other “professional” secretory cells (such as pancreatic islet cells and plasma cells) in order to maintain ER proteostasis and reduce ER stress levels.

We begin with a brief introduction to ER stress and the UPR before moving on to discuss how ameloblasts employ the UPR to cope with ER stress in both the absence and presence of genetic mutations. The evidence supporting the hypothesis that ER stress can be an etiological factor in both AI and fluorosis will be discussed along with possible therapeutic options for targeting ameloblast ER stress to ameliorate associated enamel pathologies.

## ER stress and the unfolded protein response (UPR)

The ER is responsible for trafficking all nascent proteins destined for secretion or insertion into a cellular membrane from their synthesis at the ribosome to the Golgi apparatus. The lumen of the ER contains an assortment of resident ancillary proteins (chaperones) that direct the folding of nascent polypeptides to maximize the probability that a nascent protein attains its correct functional 3-dimensional conformation. In addition, oxidoreducatase enzymes ensure that disulphide bond formation is regulated to inhibit random disulphide bond formation that if allowed could result in mis-folding. However, the molecular policing mechanisms that prevent protein mis-folding can fail. In this case, the protein may be permitted to access and become trapped in some low-energy state with a conformation that may not be biologically active or in some cases may promote pathological intracellular protein aggregation or be frankly cytotoxic (Dobson, 2003; Gregersen et al., 2006). Up to 30% of newly synthesized wild-type (WT) proteins can spontaneously mis-fold and fail to achieve their proper conformation (Schubert et al., 2000). Mutated proteins may show an even greater propensity to mis-fold and aggregate. This is the etiological basis that underpins many so-called proteopathic or conformational diseases (e.g., Parkinson's disease, Alzheimer's disease, cystic fibrosis, Huntington's disease, some cancers, diabetes and myofibrillar myopathies) (Selkoe, 2003; Lin et al., 2008; Valastyan and Lindquist, 2014; Oakes and Papa, 2015). Not surprisingly, cells have therefore evolved an active quality control system that monitors client proteins transiting through the ER. This quality control system recognizes aberrant client proteins, (whether spontaneously mis-folded WT or mis-folded mutated proteins) and acts to restore ER homeostasis thus alleviating ER stress. However, if the stress cannot be alleviated, the cell is directed toward apoptosis. The detection of mis-folded proteins is carried out by three sensor proteins that span the ER membrane: PERK, IRE1, and ATF6 (see below).

### ER stress can activate the UPR which attempts to restore proteostasis

Activation of the trans-ER membrane sensors triggers an integrated signaling pathway that initially attempts to restore ER homeostasis by: (i) reducing the secretory load, (ii) increasing the folding capacity of the ER by up-regulating chaperone expression and increasing ER volume, and (iii) increasing ER-associated protein degradation (ERAD) (Schroder and Kaufman, 2005). When ER stress is low, the sensors appear to be inactivated by their binding of GRP78 (BiP, HSPA5); present in the ER where it also functions as a chaperone, binding to cargo proteins. Mis-folded proteins in the ER lumen associate with GRP78 such that GRP78 is increasingly dissociated from the sensors, allowing them to become active and trigger the UPR (Schroder and Kaufman, 2005; Malhotra and Kaufman, 2007a). However, GRP78 may not be the main regulator in the case of sensor IRE1, which may be activated by direct interaction with mis-folded proteins (Credle et al., 2005; Gardner and Walter, 2011) that is independent of its GRP78 binding domain (Kimata et al., 2004). The initial UPR is pro-survival in nature, helping cells cope with a heavy secretory load even in the absence of any protein mutations. However, if the ER stress cannot be relieved, then the UPR, acting as a “double edged sword” (Malhotra and Kaufman, 2007b) switches to pro-apoptotic mode (Fribley et al., 2009). The decision to enter apoptosis arises through the integration of the multiple signaling outputs from the UPR; it does not involve a single event or signaling pathway (Rutkowski et al., 2006). In effect, the UPR is a graded response whose effect on the cell appears to depend on cell context, including the nature and duration of the stimulus causing the ER stress and the differential activation of the sensors under specific forms of ER stress, including their differential regulation by protein co-factors other than GRP78. The UPR is further modulated downstream allowing fine-tuning under specific circumstances (Hetz, 2012).

### The UPR in pro-survival mode—adapting to ER stress

**Figure 1:**
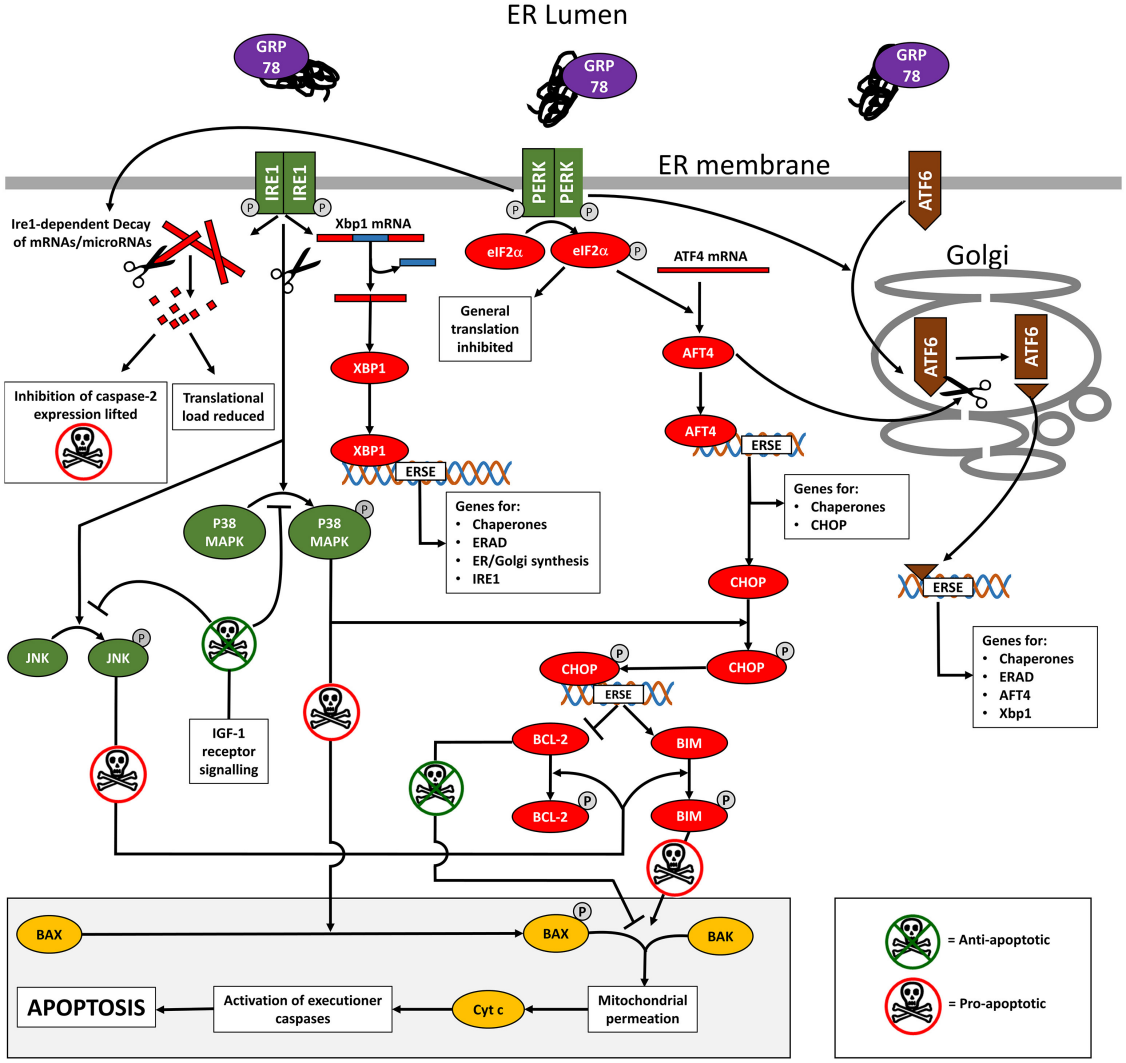
Diagram summarizing an outline of the cell signaling occurring during ER stress and the UPR. Full details are provided in the text but in brief, titration of GRP78 by misfolded proteins in the ER lumen causes phosphorylation (activation) of the ER membrane-spanning stress sensors IRE1, PERK, and AFT6. This unleashes an interconnected downstream signaling cascade of transcription factors that initially reduces the translational load by mRNA degradation and inhibition of translation. A series of ES stress response elements (ERSE) are targeted resulting in the transcription of genes that aid the cell cope with its secretory load and promote cell survival. In cases where the ER stress is unrelieved, the pro-apoptotic transcription factor CHOP is expressed. Together with the concerted activity of P38 MAPK and JNK (activated by IRE1 kinase activity), CHOP promotes apoptosis through the BAX/BAK mediated permeation of mitochondrial outer membranes. IGF-1 receptor signaling inhibits apoptosis by indirectly controlling the activation of P38 MAPK and JNK.

A detailed description of the signaling pathways triggered by ER stress is beyond the scope of this review but the role of the three sensors of the UPR in the pro-survival adaptive response will be discussed briefly below and is summarized in Figure 1.

#### IRE1

On activation, IRE1 oligomerizes and undergoes auto-phosphorylation, activating its endoribonuclease activity to drive the unconventional splicing of XBP1 mRNA (Yoshida et al., 2001; Prischi et al., 2014). Spliced XBP1 mRNA encodes a transcription factor that interacts with ER stress response elements to up-regulate UPR target genes such as chaperones (e.g., GRP78 and GRP94 (Lee et al., 2003), components of the ERAD system, genes associated with ER and Golgi expansion (lipid synthesis) (Lee et al., 2003; Ron and Walter, 2007; Hetz et al., 2011) and IRE1 itself (Tsuru et al., 2016). IRE1 also degrades specific ER-localized mRNAs (regulated Ire1-dependent decay) in an attempt to provide immediate relief from the translational load entering the already stressed ER during the initial phase of ER stress (Hollien and Weissman, 2006; Han et al., 2009).

#### PERK

On activation, PERK dimerizes and undergoes auto-phosphorylation. Activated PERK phosphorylates its downstream target, eIF2α. Phosphorylated eIF2α binds the guanine nucleotide exchange factor eIF2β, inhibiting assembly of the 43S translation initiation complex. This process effectively reduces general protein translation, thus reducing the secretory load (Harding et al., 2000). However, eIF2α-P selectively *increases* the translation of transcription factor ATF4, which targets a wide range of ER stress response elements including those involved in protein folding, assembly and metabolism (Dey et al., 2010), and as described below, apoptosis.

#### ATF6

On activation, ATF6 is trafficked to the Golgi where the cytosolic N-terminal domain is proteolytically cleaved (Ron and Walter, 2007). The cleaved ATF6 N-terminal domain is a transcription factor that translocates to the nucleus where it interacts with ER stress response elements to activate multiple UPR target genes coding elements of the ERAD system, chaperones such as GRP78 and the UPR-associated transcription factors AFT4 and Xbp1 (Yoshida et al., 2001). PERK activation enhances ATF6 synthesis and its trafficking to the Golgi for proteolytic activation which is promoted by ATF4 (Teske et al., 2011). The ATF6 pathway appears to overlap functionally to some extent with the PERK and IRE1 pathways (Wu et al., 2007).

### The UPR in pro-apoptotic mode—capitulating to ER stress

At some point during ER stress, a decision is made to abandon promotion of cell survival in favor of apoptosis. The timing of this decision, and the level of stress required to trigger the switch to pro-apoptotic mode, appear to depend on the specific nature of the stress encountered and the cell context but the decision arises following close scrutiny of the integrated signals originating from the three sensor-led arms of the UPR and the cross-talk between them as the UPR evolves (Tabas and Ron, 2011; Chen and Brandizzi, 2013; Moore and Hollien, 2015).

The IRE1 arm of the UPR appears to be the central player in directing a cell away from survival and toward apoptosis (though all arms of the UPR can activate downstream apoptotic pathways). IRE1 endoribonuclease activity can be used to manipulate the UPR response by finely controlling the degradation of specific target mRNAs (Han et al., 2009); a process enhanced when PERK is activated (Moore and Hollien, 2015), emphasizing the complex cross-talk underpinning the UPR. Under unrelieved ER stress, XBP1 mRNA splicing increases along with the regulated IRE1-dependent decay of ER-localized mRNAs encoding for ER cargo. This depletes the ER cargo including critical cell surface bound traffic (e.g., membrane bound receptors) and ER resident chaperones involved in protein folding. As a result, stress levels are tipped beyond a critical threshold, triggering the pro-apoptotic response (Han et al., 2009; Coelho and Domingos, 2014). In addition, IRE1-dependent decay targets specific microRNAs that repress the translation of caspase-2 (Upton et al., 2012), an executioner caspase implemented in apoptosis and linked to ER stress (Fava et al., 2012).

The molecular details of how the UPR finally brings about apoptosis are still the subject of intense research, driven in part by the fact that the UPR plays a pivotal role in numerous human diseases. A brief, and by no means complete, overview of the events occurring following the decision to commit to apoptosis is given below.

The transcription factor CHOP is an important UPR target gene. CHOP expression in stress-free cells is almost undetectable but it is upregulated by ATF4 (Su and Kilberg, 2008), which as described previously is itself induced following activation of PERK and AFT6 in cells undergoing ER stress (Tabas and Ron, 2011; Li et al., 2014). CHOP is post-translationally activated by phosphorylation mediated via the stress kinase p38MAPK, which is itself activated by IRE1 kinase activity. Activated CHOP up-regulates expression of pro-apoptotic gene products such as *BIM* (Puthalakath et al., 2007) whilst decreasing expression of anti-apoptotic gene products such as BCL-2 (Li et al., 2014). IRE1 kinase activity also activates a second stress kinase, JNK, which in turn can phosphorylate both BIM and BCL-2 to regulate their activity.

BIM and BCL-2 are exemplar members of a wider family of proteins that facilitate and inhibit the triggering of apoptosis respectively. In their non-phosphorylated states, BCL-2 is actively anti-apoptotic and BIM is inactive. On phosphorylation by JNK, BCL-2 loses its anti-apoptotic properties while BIM becomes actively pro-apoptotic. Under these conditions, the constitutively expressed factors BAX and BAK act in concert to trigger mitochondrial membrane disruption and the release of factors including cytochrome c that initiate the final executioner caspase cascade that leads to cell death (Nomura et al., 1999; Ow et al., 2008; Tabas and Ron, 2011). BAK is usually resident on the outer mitochondrial membrane but BAX translocates from the cytoplasm to the outer mitochondrial membrane following phosphorylation by p38MAPK or JNK (Kim et al., 2006; Ow et al., 2008), linking BAX translocation and apoptosis directly to IRE1. The regulation of BAX and BAK activity is of prime importance and it is clear even from the simplified account given above that the three arms of the UPR work in concert to tightly regulate BAX and BAK activity with numerous check points and gates in place to ensure that a point of consensus has been reached prior to committing the cell to apoptosis.

## Wild-type secretory stage ameloblasts rely on the UPR in pro-survival mode to maintain their secretory output

Specialized secretory cells, including plasma cells, pancreatic cells, hepatocytes and osteoblasts face ER stress even under normal conditions simply by virtue of their high secretory load and consequently rely on the UPR acting in pro-survival mode (Moore and Hollien, 2012). Secretory ameloblasts can be regarded as specialized secretory cells and share typical characteristics such as a prominent ER/Golgi network during the secretory stage. An early indication that the UPR is normally active in secretory stage ameloblasts was the finding that IRE was present its activated form (Kubota et al., 2005). As described in previously, a primary outcome of an active UPR is to increase the volume of ER to increase the handling capacity of the cell's secretory pathway. The volume occupied by the ER in WT pre-secretory ameloblasts increases by a factor of around 3.3 by the time the ameloblast has reached the end of the secretory stage (Tsuchiya et al., 2008). Furthermore, there is a dramatic reduction in immunohistological staining for activated phosphorylated IRE1 in maturation-stage ameloblasts compared to secretory-stage ameloblasts which is a reflection of the greatly reduced secretory load transiting the ER in maturation-stage ameloblasts (Tsuchiya et al., 2008). These authors also reported that expression of spliced Xbp1 mRNA was five times greater in secretory enamel organ cells compared to maturation stage enamel organ, indicating that IRE1 activation had indeed triggered the UPR in the secretory ameloblasts (Tsuchiya et al., 2008). These data are important as they indicate that WT secretory ameloblasts are stressed by their secretory load under normal conditions and require the UPR to help manage the situation. The prominent client protein in the secretory ameloblast pathway is amelogenin; a hydrophobic protein that is well known for its propensity to self-assemble/aggregate. Molecular cross-linking studies showed that amelogenin begins to self-assemble during its transit through the ameloblast secretory pathway (Brookes et al., 2006) and the regulation of these intramolecular interactions may well require sustained input from the folding machinery under the influence of UPR signaling.

As described previously, CHOP expression is a marker for UPR-induced apoptosis and is undetectable in stress-free cells. Despite evidence that the UPR operates in pro-survival mode in the secretory stage of amelogenesis, *Chop* expression was detected in secretory stage enamel organs of WT mice by quantitative PCR (Brookes et al., 2014) suggesting that even WT ameloblasts may be on their way toward an apoptotic end-point. It was not clear from this ensemble data whether the level of *Chop* expression represented relatively low level expression in all ameloblasts or whether it reflected relatively high expression in a sub-population of cells. Why is it that WT secretory ameloblasts do not succumb to apoptosis given that they appear to be expressing *Chop* especially as rodent WT ameloblasts have been described as “hard wired” for apoptosis (Joseph et al., 1999)? It has been proposed that activation of IGF-1 receptors, expressed by ameloblasts throughout amelogenesis, except for a short window during the transition from the secretory to maturation stage (Joseph et al., 1994), inhibits pro-apoptotic events by modulating BCL-2 and BAX or by directly inhibiting caspase 3 activation (Joseph et al., 1999). These events are downstream of CHOP (see Figure 1) so that even when CHOP is expressed, IGF-1 signaling may prevent the final commitment to apoptosis. Later work confirmed that IGF-1 receptor activation inhibits JNK and P38 MAPK activation (Galvan et al., 2003), both of which are involved in committing the cell to apoptosis by modulating BCL-2, BIM and BAX phosphorylation at a point downstream of CHOP expression (see Figure 1). We can therefore hypothesize that the UPR assists WT secretory ameloblasts to cope with their heavy secretory load but also primes the most stressed cells for apoptosis while IGF-1 signaling prevents the cells from taking the final steps that commit to apoptosis. An estimated 25% of ameloblasts abruptly succumb to apoptosis at the end of the secretory stage (Smith and Warshawsky, 1977). We hypothesize that the most stressed secretory stage ameloblasts are prevented from undergoing apoptosis by IGF-1 signaling during secretion, but, during the transition from secretion to maturation, when IGF-1 receptor expression ceases, the brake on apoptosis is released and pre-disposed ameloblasts rapidly undergo apoptosis. However, as IGF-1 receptor expression increases again once ameloblasts enter the maturation stage proper (Joseph et al., 1994), the brake on ameloblast apoptosis is re-applied. IGF-1 signaling also up-regulates the expression of the chaperone GRP78 independently of the UPR which enhances the folding capacity of the ER and provides additional protection against ER stress (Novosyadlyy et al., 2008). The importance of IGF-1 in amelogenesis is emphasized by the fact that ameloblasts express IGF-1 in addition to the IGF-1 receptor, establishing an autocrine signaling loop (Joseph et al., 1996).

Given that WT secretory ameloblasts are already using the UPR to help cope with ER stress generated by their demanding secretory function, the cells are already on a path that can lead to an apoptotic end-point. This raises the question as to whether there is a role for the ameloblast UPR where enamel matrix proteins (or others) are affected by genetic mutations and generate even more intense levels of ER stress. The net effects of the UPR in the presence of an enamel protein mutation may be relatively mild (e.g., due to reduced translation of enamel proteins) or more severe (e.g., due to ameloblast apoptosis). The next section will review the evidence that mutations in enamel matrix proteins can indeed cause AI driven by ER stress.

## ER stress and the UPR as an etiological factor in AI

Classically, AI has been approached from the perspective that mutations in genes encoding for secreted enamel matrix proteins would impact on protein functionality and behavior within the extracellular matrix itself. For example, studies have shown that certain AMELX mutations can affect amelogenin self-assembly (Paine et al., 2002; Zhu et al., 2011) and the ability to adsorb onto hydroxyapatite and control crystal growth (Zhu et al., 2011). It is extremely likely therefore that dysfunction of mutated enamel proteins in the extracellular compartment can drive AI. However, in recent years it has become clear that in some specific cases, AI may be associated with the intracellular phenomenon of ameloblast ER stress and UPR activation.

The first evidence that ER stress could drive AI came from detailed phenotyping of a mouse model exhibiting an *Amelx*^*p.(Y64H)*^ mutation (Barron et al., 2010; Brookes et al., 2014). More recently mouse *Enam*^*p.(S55I)*^ and human *ENAM*^*p.(L31R)*^ mutations have also been associated with ameloblast ER stress (Brookes et al., 2017). ENAM is expressed at very low levels compared to AMELX and is biochemically distinct—in mice being over seven times the molecular weight of AMELX and more basic in nature (Hu et al., 1998). The two proteins may have originated from a common ancestral gene (Sire et al., 2006) but no longer share sequence homology, suggesting divergent functional roles. It is therefore somewhat surprising that enamel from female mice heterozygous for *Amelx*^*p.(Y64H)*^ (there is no AMELY transcript in rodents) closely phenocopies mice heterozygous for *Enam*^*p.(S55I)*^.

**Figure 2:**
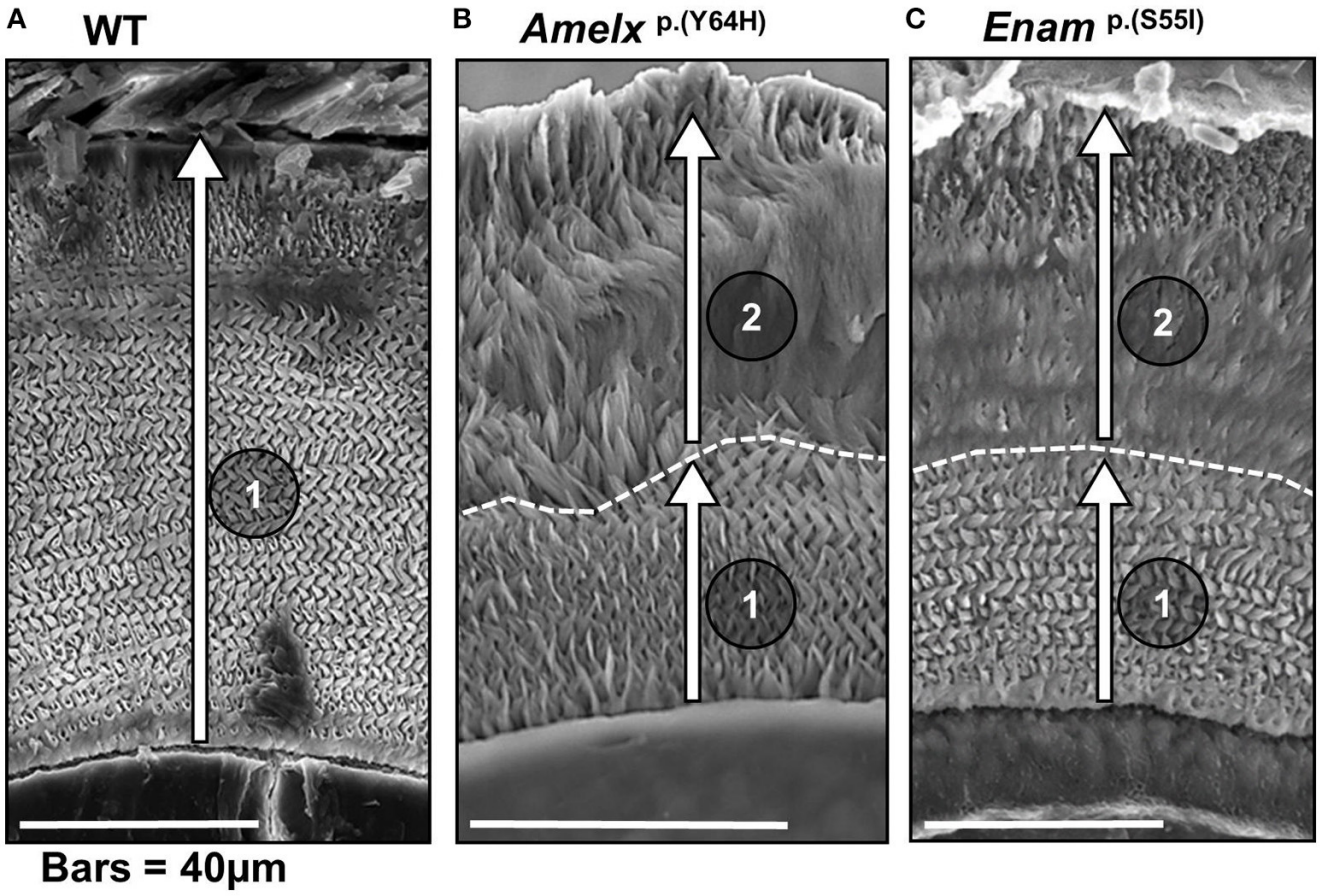
Scanning electron micrographs of transverse sections through mandibular incisors of **(A)** WT mice, **(B)** mice heterozygous for the *Amelx* ^*p.Y64H*^ mutation and **(C)** mice heterozygous for the *Enam*^*p.S55I*^ mutation at or near the point of eruption (previously reported by Brookes et al., 2014, 2017). **(A)** The incremental secretion of the inner enamel (in the direction indicated by arrow 1) by WT ameloblasts produces the decussating prismatic architecture characteristic of rodent enamel. **(B)** In mice heterozygous for Amelx^*p.Y64H*^, the ameloblasts begin the incremental secretion of an apparently structurally normal layer of enamel (arrow 1). However, after about 40 μm of enamel is secreted, the ameloblasts continue to secrete enamel (arrow 2) but they lose the ability to produce the characteristic decussating prismatic architecture. **(C)** In mice heterozygous for Enam^*p.S55I*^, the ameloblasts similarly secrete a structurally normal layer initially (arrow 1) but enamel secreted thereafter is structurally abnormal (arrow 2). We hypothesize that the UPR, initially acting in pro-survival mode, supports normal ameloblast function and allows the ameloblasts to produce an inner layer of decussating enamel. Later, the UPR switches to a more draconian mode and ameloblast function is perturbed resulting in the secretion of structurally abnormal outer layer of enamel. The dotted line marks demarcation between the inner decussating and outer non-decussating enamel. **(B,C)** were derived under Creative Commons CC BY licenses 3.0 and 4.0 (https://creativecommons.org/licenses/) respectively from: Brookes et al. (2014), and phenotypic rescue using 4-phenylbutyrate; and Brookes et al. (2017). Both published by Oxford University press.

Incisor enamel from heterozygous mice of both genotypes revealed an unusual phenotype in which the first 30–50 μm of initially secreted incisor enamel (i.e., the inner enamel adjacent to the dentine) exhibited the decussating prismatic structure characteristic of WT rodent incisor enamel but this was overlaid with subsequently secreted, structurally abnormal, aprismatic enamel (Brookes et al., 2014, 2017). This shared structural phenotype suggests a common underlying etiology despite the presumed functional differences between AMELX and ENAM. Secretory-stage ameloblasts of both genotypes exhibited abnormal retention of enamel matrix proteins, indicating a compromised secretory pathway. Notably, ameloblasts in both *Amelx*^*p.(Y64H)*^ and *Enam*^*p.(S55I)*^ animals showed a clear upregulation of markers indicative of ER stress and an activated UPR (e.g., *Grp78, Xbp1, Grp94*, and *Atf4*). During the early secretion stage, ameloblasts in affected mice were present as an ordered monolayer characteristic of WT animals and produced a structurally normal inner layer of enamel. However, in the later stages of secretion, the ameloblast monolayer became more disorganized, coincident with the loss of normal prismatic structure. These observations were interpreted in terms of the UPR initially acting in pro-survival mode, which maintained a functional ameloblast monolayer and allowed the cells to produce a structurally normal initial layer of enamel in both mutant genotypes. However, as the UPR signal evolved, ameloblasts from female mice expressing the *Amelx*^*p.(Y64H)*^ (comprising ~50% of the total ameloblast population due to random X-chromosome deactivation) were directed toward apoptosis as evidenced by increased *Chop* expression, which severely disrupted the ameloblast monolayer leading to the production of structurally abnormal enamel. In contrast, *Chop* expression in enamel organs from mice heterozygous for *Enam*^*p.(S55I)*^ remained at WT levels and it was assumed that the evolving UPR in these animals was up-regulated but failed to reach the tipping point required to trigger apoptosis. Nevertheless, the response was evidently sufficient to compromise the integrity of the ameloblast monolayer which resulted in the production of a structurally abnormal outer layer of enamel. This section is summarized in Figure 2.

Secretory stage ameloblasts in hemizygous male mice carrying the *Amelx*^*p.(Y64H)*^ mutation and mice homozygous for *Enam*^*p.(S55I)*^ exhibited increased expression of the pro-apoptotic transcription factor *Chop* compared to WT animals. Animals of both mutant genotypes did not produce a recognizable enamel layer. The failure to produce any enamel may have been related to the fact that the UPR evolved more quickly away from a pro-survival mode. However, another possibility is that insufficient mutated AMELX or ENAM molecules were secreted into the matrix to support amelogenesis and even if they were, the mutation could have impacted on their extracellular function.

What about the anti-apoptotic role of IGF-1 in these animals, which should be unaffected by mutations in the *Amelx* and *Enam* genes? We can only assume that if the ER stress reaches a critical intensity, the UPR can circumvent the “protection” provided by IGF-1 receptor signaling. The details of how this can be achieved are unclear but it may be simply due to the significant increase in *Chop* expression in the presence of a mutation that overcomes the anti-apoptotic effects of IGF-1 receptor signaling.

Finally, is there any evidence that ER stress and the UPR are etiological factors in human AI? It is virtually impossible to study amelogenesis in humans due to the obvious issues in obtaining fresh tooth germs to study. However, due to the incremental nature of enamel secretion, mature enamel can provide a temporal record of events that occur during amelogenesis. This is exemplified by the enamel phenotype for mice heterozygous for *Amelx*^*p.(Y64H)*^ and *Enam*^*p.(S55I)*^ described above. A similar enamel phenotype was recently described in teeth obtained from an AI patient heterozygous for an *ENAM*^*p.(L31R)*^ mutation. The mature enamel phenotype from the patient's exfoliated teeth exhibited the same layer of structurally normal inner enamel overlaid by a subsequently secreted, structurally abnormal enamel outer layer (Brookes et al., 2017) comparable to that described in the mouse models. This is not unequivocal evidence that the UPR was responsible for AI in this patient but the developmental record remaining in the enamel indicates that ameloblast function was severely affected after an initial period of near-normal secretory activity, reminiscent of an evolving UPR.

## The role of the UPR in the pathobiology of fluorosis

Early indications that excess fluoride may trigger an ER stress–like response in ameloblasts were provided by histological reports suggesting that fluoride disturbs ameloblast intracellular protein trafficking (Matsuo et al., 1996), including the retention of ER cargo and the appearance of a dilated ER (Hassunuma et al., 2007). These observations are consistent with ameloblasts suffering ER stress but the up-regulation of UPR components provides unequivocal evidence that the UPR is associated with the ameloblast response to fluoride. The first such evidence suggested that fluoride promoted IRE1 activation in maturation stage ameloblasts *in vivo* and up-regulated *BiP (Grp78), Xbp-1*, and *Chop* expression in the LS8 ameloblast cell line (Kubota et al., 2005). The notion that fluoride may impair protein secretion was further supported when LS8 cells were transfected with secreted alkaline phosphatase. Fluoride decreased the secretion of the phosphatase in a dose-dependent manner while intracellular levels of phosphatase were concomitantly increased, along with increased levels of activated PERK, phosphorylated eIF2α, and BiP (GRP78) (Sharma et al., 2008). Later studies using LS8 cells additionally reported that the third stress sensor, ATF6, is also activated by fluoride (Wei et al., 2013). Increasing levels of phosphorylated eIF2α were also seen in maturation-stage ameloblasts in mice provided with increasing concentrations of fluoride in their drinking water (Sharma et al., 2008, 2010). However, no increase in levels of phosphorylated eIF2α was seen in secretory-stage ameloblasts exposed to fluoride in the same study. The differential effect of fluoride on secretory stage ameloblasts may be explained by the acid hypothesis for fluorosis, in which periodic falls in enamel matrix pH during the maturation stage (but not the secretory stage) lead to protonation of F^−^ to HF, which greatly increases its ability to diffuse across cell membranes and so enter the cytoplasm. At cytoplasmic pH, HF dissociates, resulting in a HF concentration gradient across the cell membrane. Trapped cytoplasmic F^−^ would then continue to accumulate intracellularly to levels that trigger a pathological response (Sharma et al., 2010).

The possibility that fluoride can disrupt the intracellular secretory pathway could lead us to conclude that this mechanism alone triggers the three UPR sensors. However, a known cytotoxic effect of fluoride is its ability to promote the accumulation of reactive oxygen species (ROS) by inhibiting free radical scavenging systems such as those based on glutathione peroxidase and superoxide dismutase (Chlubek, 2003). ROS are normally generated by a variety of cellular processes including mitochondrial electron transport and as a byproduct of disulfide bond formation in the lumen of the ER (Santos et al., 2009). Inability to deal with ROS could lead to redox imbalance in the ER, a situation that can trigger the UPR via the three ER stress sensors and/or via ROS-promoted calcium efflux (Eletto et al., 2014). Evidence that fluoride triggers oxidative stress in ameloblasts was provided by the observation that UCP-2 (an electron transport uncoupler that provides an adaptive defense against oxidative stress; Moukdar et al., 2009), was up-regulated in mice drinking fluoridated water (Suzuki et al., 2014). These authors suggested that in addition to the effect of fluoride on the UPR in maturation stage ameloblasts, their function may be further compromised by energy deficiency caused by the impact of UCP-2 activity uncoupling electron transport from ATP synthesis which could impact on crucial maturation stage processes that require energy, such as the active transport of mineral ions.

Once activated, how does the UPR actually effect extracellular events in the maturation stage enamel matrix under fluorotic conditions? Fluorosis is associated with enamel hypomineralization and abnormal retention of secretory stage matrix proteins in maturation stage enamel, where they could then inhibit secondary crystal growth (Den Besten, 1986; Smith et al., 1993). Under normal circumstances, KLK4, secreted during the maturation stage, degrades residual secretory stage matrix proteins but fluoride does not directly inhibit either KLK4 or its activator proteases (including MMP20) (Tye et al., 2011). Instead, it appears that fluoride inhibits protein expression in maturation stage ameloblasts which reduces the amount of KLK4 available to degrade the residual secretory stage enamel matrix. As described previously, phosphorylation of eIF2α, by PERK during the UPR, decreases general protein translation (Section PERK) and its phosphorylation on exposure to fluoride in rat maturation stage ameloblasts was shown to downregulate KLK4 expression, whereas secretory stage expression of AMELX, AMBN, and MMP20 were unaffected (Sharma et al., 2010). This prompted the suggestion that fluoride reduced KLK4 expression and prevented the efficient degradation and removal of residual secretory stage matrix proteins leading to a pathological retention of protein in the maturation stage tissue. It has been suggested that enamel matrix proteins could have a higher affinity for fluorotic enamel crystals (Tanabe et al., 1988) which could further compromise the removal of residual matrix from maturation stage tissue where KLK4 levels are already depleted.

**Figure 3:**
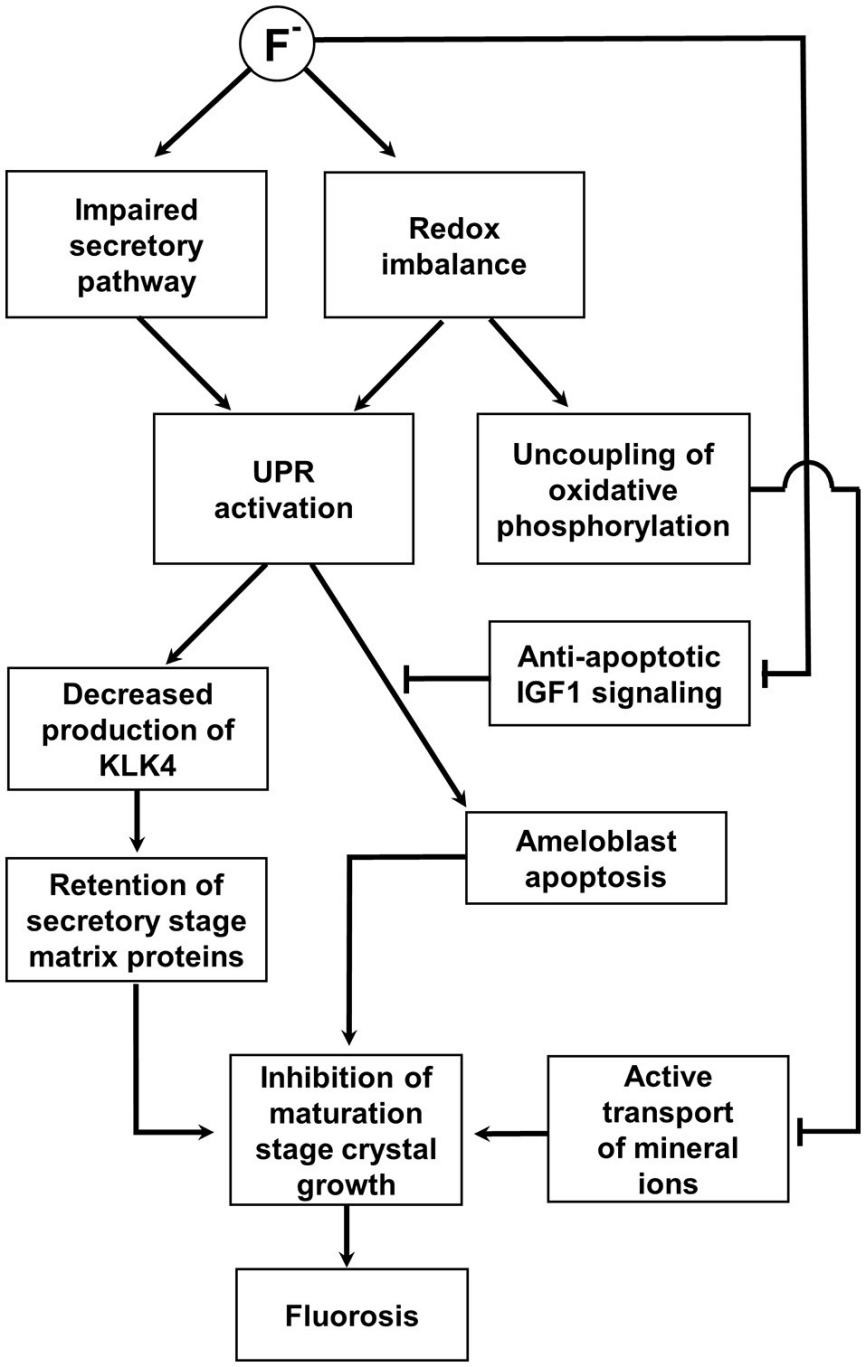
Schematic diagram summarizing the hypothesized role of the UPR in fluorosis as described in the text. This impairs the secretory pathway and promotes a redox imbalance; both can potentially activate the UPR. The UPR decreases the production of KLK4 and degradation and removal of the secretory stage matrix is compromised. Retention of the secretory matrix then inhibits maturation stage crystal growth. In addition, mitochondrial oxidative phosphorylation is uncoupled from electron transport in an attempt to restore redox balance. The resulting reduction in ATP synthesis would limit the active transport of mineral ions into the matrix further compromising maturation stage crystal growth. A severe fluoride challenge induces apoptosis and it is hypothesized that this would be mediated by the UPR. Apoptotic signaling under these circumstances may well be unchecked by anti-apoptotic IGF1 signaling as IGF1 expression is reduced by fluoride. See main text for references.

One final consideration in relation to fluorosis pathobiology and the role of the UPR is the potential effect of fluoride on the expression of IGF-1. Since anti-apoptotic IGF-1 signaling appears to be an important pathway in amelogenesis, it is interesting that when primary mouse osteoblast cultures were treated with fluoride, the resulting oxidative stress led to reduced IGF-1 expression and increased apoptosis (Wang et al., 2011). Maturation-stage ameloblasts undergo apoptosis under high fluoride regimes (Kubota et al., 2005) and it is possible that fluoride not only triggers an apoptotic UPR response but further enhances that response by simultaneously degrading the anti-apoptotic effects of IGF-1. This section is summarized in Figure 3.

## Targeting ER stress and the UPR as a therapeutic intervention in enamel pathologies

ER stress and an up-regulated UPR are now recognized as etiological factors in numerous serious human diseases (Oakes and Papa, 2015). Collectively, these diseases can be classed as proteopathies, or conformational diseases, where protein mis-folding and aggregation causes loss of ER homeostasis leading to an up-regulated UPR. Much work is ongoing to find therapeutic strategies to combat proteopathic disease. In general, this is based on identifying molecules (synthetic chaperones) that can prevent protein mis-folding and restore normal protein trafficking, prevent mis-folded proteins activating the three transmembrane ER stress sensors or modulate the UPR to inhibit apoptosis. A typical example where ER stress has been targeted therapeutically is progressive familial intrahepatic cholestasis type 2 caused by a p.T1210P mutation in the canalicular bile salt export pump (BSEP). In cultured cells transfected with *BSEP*^*p.T1210P*^, the BSEP ^*p.T1210P*^ protein was retained in the ER, impeding its transportation to the canalicular membrane. Addition of the synthetic chaperone, 4-phenylbutyrate (4-PB), partially corrected the situation. A child homozygous for the *BSEP*^*p.T1210P*^ mutation was treated with oral 4-PB with a subsequent improvement in liver function and partial restoration of biliary bile acid secretion. In this case, it appeared that 4-PB restored the trafficking of the mutated bile salt export pump to the canalicular membrane, where, despite the mutation, it was still functional to some degree. Restoring ER trafficking in this case allowed the mutated protein to escape the ERAD system (Gonzales et al., 2012). 4-PB was also able to prevent the aggregation of four different myocilin mutants in the ER of transfected cells and restore the secretion of mutant myocilin. This rescued the cell from ER stress and significantly reduced apoptosis in the transfected cells, leading the authors to propose that 4-PB could be used as a therapeutic agent to treat blindness causing primary open-angle glaucoma (Yam et al., 2007). Topical application of 4-PB eye drops in mice was later shown to restore the secretion of mutant myocilin and return intraocular pressure to WT levels (Zode et al., 2012). In addition to its ability to interact with mis-folded or aggregated proteins in the ER lumen, 4-PB can also influence gene expression by its activity as a histone deacetylase inhibitor that also inhibits the deacetylation of a range of transcription factors, including NF–κβ. This in turn indirectly affects the expression of numerous target genes including those associated with the anti-apoptotic response (Ryu et al., 2005). In short, 4-PB can influence the transcription of wide range of genes by influencing the epigenetic control of gene expression and the covalent regulation of transcription factor activity. This may explain its reported ability to moderate the UPR and UPR-mediated apoptosis (Vilatoba et al., 2005; Basseri et al., 2009; Yue et al., 2016).

Despite this, the specific cellular response to 4-PB depends on cell context. For example, 4-PB can be pro-apoptotic in myeloid leukemia cells (DiGiuseppe et al., 1999) and prostate cancer cells (Melchior et al., 1999). The question as to whether 4-PB might have therapeutic value in treating AI was investigated when female mice heterozygous for the *Amelx*^*p.(Y64H)*^ mutation were fed 4-PB in their diet. A dramatic rescue of the AI phenotype resulted (Brookes et al., 2014). In this context, 4-PB did not restore AMELX secretion as might be expected were it to be acting as a synthetic chaperone. Instead, 4-PB appeared to inhibit apoptosis in the 50% of ameloblasts expressing *Amelx*^*p.(Y64H)*^. This presumably allowed the remaining ameloblasts expressing WT AMELX to complete amelogenesis. It is possible that AMELX synthesis and secretion was increased in the unaffected ameloblasts and that this compensated for the fact that only half the ameloblasts were secreting matrix.

It is currently unknown whether or not 4-PB treatment can rescue the enamel phenotype in cases of AI other than that in female mice heterozygous for the *Amelx*^*p.(Y64H)*^ mutation. However, restoring a stalled secretory pathway or inhibiting the pro-apoptotic actions of the UPR will not rescue the phenotype if the mutated protein in question is dysfunctional when it is secreted into the extracellular matrix.

The suggestion that 4-PB is anti-apoptotic in stressed ameloblasts is further supported by the report that apoptosis triggered by exposure to fluoride in an ameloblast-derived cell line was inhibited by 4-PB, resulting in an anti-apoptotic BCL2/BAX ratio (Suzuki et al., 2017). However, 4-PB was unable to completely prevent fluorosis in mice drinking fluoridated water though it did improve some aspects of the condition compared to control animals who did not receive 4-PB. The failure of 4-PB to rescue fluorosis could be explained if its effect was limited to inhibiting apoptosis whilst having no influence over the effect of fluoride in downregulating KLK4 expression.

An alternative option to treat fluorosis would be to target the oxidative stress that is triggering the UPR in the first place. Numerous studies have shown that antioxidants can counter the oxidative damage caused by fluoride in bone and soft tissues. For example, fisetin, an anti-oxidant polyphenol flavonoid, protects against fluoride-induced oxidative damage in osteoblast cell lines (Inkielewicz-Stepniak et al., 2012) and pretreating rats with the flavonoid silymarin protected against fluoride-induced oxidative stress in the brain. In the case of enamel, Suzuki et al. (2014) reported that a diet enriched with the antioxidant vitamin E had no protective effect against enamel fluorosis in mice drinking 50 ppm fluoride. More recently, it was reported that the antioxidant carotenoid lycopene inhibited fluoride induced ameloblast apoptosis and enamel fluorosis in rats by combating oxidative stress (Li et al., 2017). However, the efficacy of such agents against enamel fluorosis in humans remains unknown.

## Future perspective

The phenotypic rescue of female mice heterozygous for *Amelx*^*p.(Y64H)*^ proves the principle that AI driven by ER stress and a pro-apoptotic UPR can be treated therapeutically with 4-PB. 4-PB is an approved therapeutic for urea cycle disorders. Acting as an excretable ammonium scavenger, 4-PB is administered orally in high doses from birth. However, 4-PB is contraindicated during pregnancy and so its therapeutic value would be restricted to protecting those permanent teeth whose enamel begins to mineralize after birth.

Clearly, more research is required using relevant mouse models to establish how common ER stress is as an etiological factor in human AI and how appropriate is targeting ER stress and the UPR as a treatment option. There are numerous other compounds under investigation for their therapeutic potential in terms of influencing folding of mutated proteins, inhibiting aggregation of mutated proteins and modulating the UPR. (Schonthal, 2012; Denny et al., 2013) and these may be more effective than 4-PB for treating specific cases of AI depending on the mutation involved. To treat AI in patients with such compounds, it would first be necessary to establish that ER stress was involved in the etiology in each case and then identify the most effective therapeutic agent able to combat the effects of the specific mutation involved in a personalized medicine approach using cell models.

In summary, ER stress and the UPR play an important role in maintaining ameloblast function and proteostasis under high secretory load during amelogenesis. We also know that it plays a role in the etiology of enamel pathology, and that, at least in some cases, AI can now be added to the growing list of proteopathic diseases. Proteopathies include several of the major diseases of our age and there is intense research underway to identify compounds of therapeutic value. It is an exciting possibility that anti-proteopathic drugs may provide an effective treatment option in amenable cases of AI. Fluorosis is not a classic proteopathic disease but the etiological involvement of the UPR raises the possibility that drugs that can modulate the UPR, or control the oxidative stress triggering the UPR, may be of therapeutic value in areas where fluorosis is endemic.

## Author contributions

SB, MB, MD, and JK contributed to the writing of the manuscript and its final approval. All authors agree to be accountable for all aspects of the work in ensuring that questions related to the accuracy or integrity of any part of the work are appropriately investigated and resolved.

### Conflict of interest statement

The authors declare that the research was conducted in the absence of any commercial or financial relationships that could be construed as a potential conflict of interest.
